# LEI: A Novel Allele Frequency-Based Feature Selection Method for Multi-ancestry Admixed Populations

**DOI:** 10.1038/s41598-019-47012-y

**Published:** 2019-07-31

**Authors:** Michael J. Wathen, Yadu Gautam, Sudhir Ghandikota, Marepalli B. Rao, Tesfaye B. Mersha

**Affiliations:** 1 Division of Biostatistics and Epidemiology, University of Cincinnati, Cincinnati, OH 45229 USA; 2 Department of Pediatrics, Cincinnati Children’s Hospital Medical Center, University of Cincinnati, Cincinnati, OH 45229 USA; 3 Department of Computer Science, University of Cincinnati College of Engineering, Cincinnati, OH 45220 USA

**Keywords:** Statistical methods, Population genetics

## Abstract

Next-generation sequencing technologies now make it possible to sequence and genotype hundreds of thousands of genetic markers across the human genome. Selection of informative markers for the comprehensive characterization of individual genomic makeup using a high dimensional genomics dataset has become a common practice in evolutionary biology and human genetics. Although several feature selection approaches exist to determine the ancestry proportion in two-way admixed populations including African Americans, there are limited statistical tools developed for the feature selection approaches in three-way admixed populations (including Latino populations). Herein, we present a new likelihood-based feature selection method called Lancaster Estimator of Independence (LEI) that utilizes allele frequency information to prioritize the most informative features useful to determine ancestry proportion from multiple ancestral populations in admixed individuals. The ability of LEI to leverage summary-level statistics from allele frequency data, thereby avoiding the many restrictions (and big data issues) that can accompany access to individual-level genotype data, is appealing to minimize the computation and time-consuming ancestry inference in an admixed population. We compared our allele-frequency based approach with genotype-based approach in estimating admixed proportions in three-way admixed population scenarios. Our results showed ancestry estimates using the top-ranked features from LEI were comparable with the estimates using features from genotype-based methods in three-way admixed population. We provide an easy-to-use R code to assist researchers in using the LEI tool to develop allele frequency-based informative features to conduct admixture mapping studies from mixed samples of multiple ancestry origin.

## Introduction

Genome sequencing and genotyping technologies have generated millions of single nucleotide polymorphism (SNP) data capturing vast amounts of the genetic variations that characterize the individual differences in human populations including admixed ancestry proportions and continental origins in humans^1^. Such advances in high throughput technology enable us to apply multi-locus genomic data and estimate individual ancestry as a continuous trait instead of using self-reported binary racial groups. Knowledge of genetic ancestry of individuals in the sample beyond the self-reported race/ethnicity is warranted in many applications of genomic data including disease-gene mapping via case-control association or admixture mapping. Ancestral heterogeneity among the samples in a case-control association studies can induce spurious associations; hence the accurate characterization of the study population is needed in order to detect the true genetic association^2–4^. On the other hand, the admixture mapping methods are built upon the accurate comparison of the individual’s ancestry in specific genomic region with the individual genome proportion. Thus, the knowledge of individual genome proportions of an admixed population is critically relevant for assigning individuals to their continental ancestry in association mapping as well as mapping the risk loci using admixture mapping^5,6^.

The task of identifying and assigning an individual’s genomic regions to the correct ancestries can be difficult in admixed populations with multiple ancestral origins. It is estimated that 80–90% majority of the common genetic variants are shared among individuals globally, hence, are less informative of continental origins^7,8^. Though rare genetic variants are more likely to be restricted within a continental population and thus more informative of ancestry^9^, such variants are rarely captured or genotyped in study samples. Hence, prioritizing ancestry informative markers (AIMs) among the common genetic variants is of paramount importance in studies of population structure and disease genetics^10^. The number of markers required for the population assignment will depend on the population under consideration, its respective level of genetic differentiation, and on the desired stringency of the assignment^11^.

AIMs are SNPs that exhibit large variation in minor allele frequencies (MAF) among reference populations. Carefully selected AIM panels can delineate population structure efficiently by detecting variation in individual ancestry that can confound association analyses and forensic investigations in increasing false positive results or reducing power^12^. Although several methods exist to identify AIMs in admixed populations, specifically from two-ancestral groups, there are limited statistical tools to develop informative markers for three-way admixed populations, including Latino populations^1,11,13^. Current approaches for identifying AIMs in three-way admixed population such as Latino, utilize pairwise marker informative measures such as Delta or F_ST_, and integrate the pairwise AIMs to define the multi-way AIMs^14^. Alternately, machine learning approaches such as Principal Components Analysis (PCA), Random Forest (RF), and Support Vector Machine (SVM) can be trained on a reference panel and evaluated post-hoc to perform feature selection. As a feature selection approach, these methods can be used to rank the informative SNPs, which serve as a classifier to discriminate populations based on continental ancestry. However, all these methods require the individual-level genotype data and the selection of subsets of informative SNPs from ever-increasing genomic datasets with over millions of SNPs can be very expensive^13,15^. Additionally, individual level data are often unavailable or restricted for public use. It is thus desirable to find an efficient feature selection method, which not only identifies the AIMs to estimate the admixture proportions in samples from admixed population with high accuracy, but is also computationally feasible, cost-effective, and applicable to multi-way admixture and summary level data.

This study aims to develop an efficient algorithm and a statistical tool to develop informative markers for three-way admixed populations, including Latino populations. In the present work, we introduce a maximum likelihood estimator, which was named as the Lancaster Estimator of Independence (LEI) after O. E. Lancaster who developed an estimator for probability distributions in 1969^16^. Here, we extend the LEI application into genomics as a measure of marker informativeness with the unique ability to compare multiple ancestral populations using allele frequency summary statistics.

In implementing the LEI, we first construct an algorithm to extract ancestry informative marker subsets that accurately classify subject ancestral population membership using both individual level genotype data and summary level allele frequency data. We compare the performance of LEI-based approaches with standard machine learning approaches applicable for feature selection in three-way admixed population, including Principal Components Analysis (PCA), Random Forest (RF), and Support Vector Machine (SVM). Each feature selection approach is built upon by scoring SNPs that reflect their ability to classify the ancestral populations. Using the top ranked SNPs from each approach, we first compared the classification accuracy at continental level and showed that the allele frequency based LEI approaches performed equally with genotype based feature selection approaches for classifying 2-way and 3-way continental populations from the 1000 Genomes Project^9^. Next, we compared performance of top-ranked features from LEI-based approaches for estimating the ancestry proportion of admixed individuals in 2-way and 3-way admixture simulated data. Finally, we prepared an R code for the LEI method. The R-source code is freely available to download and included in the Appendix.

The availability of cheap genotyping and sequencing technology generated millions of data points, which makes it difficult to utilize the entire genotype data for ancestry inference especially for multi-ancestry admixed populations. As the summary level data becomes more accessible, LEI provides a methodological advancement in the feature selection process with wide applicability in multi-way admixture analysis which will become more common due to global admixture.

## Materials and Methods

### Data sets

#### Continental ancestry real datasets

To study the continental ancestry, we used the 1000 Genomes Phase 3 release with a total of 310 subjects belonging to the following populations: CEU (Utah residents with Northern and Western European ancestry: n = 99), CHB (Han Chinese in Beijing: n = 103), and YRI (Yoruba in Ibadan, Nigeria: n = 108)^9^. Pre-analysis data cleaning and formatting were performed using PLINK^17^.

#### Admixed ancestry simulated datasets

To study the admixture ancestry, we simulated two different admixture scenarios - one for two-way admixture and the other for three-way admixture, using the reference populations of CEU (n = 113), YRI (n = 113), and CHD (Chinese in metropolitan Denver, Colorado, USA; n = 85) from the HapMap phase III^18^. For the two-way admixture scenario, we chose the two reference populations - CEU and YRI samples and for the three-way admixture simulation, we further incorporated the CHD population with CEU and YRI populations. Our motivation behind the selection of CHD instead of CHB is that CHD is more plausible to get admixed in the future in mainland US. The number of samples and SNPs investigated were kept constant for both admixture types. The performance of the feature selection methods (i.e., LEI, PCA, RF, and SVM) were evaluated using the root mean square error (RMSE) computed from the inferred and true ancestry.

For the two-way admixture simulation, we randomly selected 200 chromosomes from CEU and YRI. Then, 200 admixed chromosomes were created as a mosaic of randomly selected pair of CEU and YRI haplotype. We followed the simulation strategy similar to the method used in HAPMIX^19^. The admixture proportion of YRI from each chromosome was randomly generated from $Beta(\alpha =12,\,\beta =3)$ distribution, which resulted in an average of 80% YRI proportion. To initiate the admixture process, we randomly selected one chromosome from CEU and one chromosome from YRI. Then, we randomly started with choosing the first marker from CEU or YRI chromosome with probability ${\theta }_{1}\, \sim \,Beta(\alpha =12,\,\beta =3)$. For the next marker, the ancestry was resampled with the Poisson probability $1-{e}^{-gl}$, where g is the number of generations since the admixture, and l = base pair difference/10^8^ (approximates the genetic distance between the two markers). If the ancestry was found to switch, we chose the next allele from YRI with *θ*_1_ probability. We simulated all the 20,085 markers in chromosome 22. The 200 simulated admixed chromosomes were randomly paired to generate 100 simulated admixed individuals. We used g = 8 in our two-way simulation which roughly matches the number of generations of admixture in the African American population^20,21^.

For the three way-simulation, we fixed the admixture proportion of CEU, YRI, and CHD to 60%, 30% and 10%, respectively. We randomly selected one chromosome from each of the CEU, YRI, and CHD panels. We simulated admixed chromosomes by randomly selecting a segment of 100,000 base pairs from CEU, YRI, or CHD with probability (0.6, 0.3, 0.1). We simulated 200 chromosomes and randomly paired to generate 100 simulated admixed individuals.

### Feature selection methods

In this study, we refer feature selection as a process of finding the most informative subset of SNPs from a large panel of SNPs. The selected set of SNPs can be further used for downstream analysis such as population classification or ancestry inference. Feature ranking is one of the frequently used criteria in many feature selection methods that apply one or more ranking scores to separate the highly relevant features from the least relevant features^22^. In this study, we present five different measures of marker informativeness as the feature ranking criteria and compare their performance in three-way population classification and ancestry inference.

#### Lancaster estimator of independence (LEI)

We first define LEI between two categorical variables X and Y. Let $X=({x}_{1},\,{x}_{2},\ldots ,{x}_{m})$ and $Y=({y}_{1},\,{y}_{2},\ldots ,\,{y}_{k})$, with joint probability distributions, ${p}_{ij}=P(X={x}_{i},\,Y={y}_{j})$, *i* = *1, 2, …, m* and *j* = *1, 2, …, k*. Then:

1 $${\theta }^{2}={\sum }_{i,j}\frac{{p}_{ij}^{2}}{{p}_{i+}{p}_{+j}}-1,$$

where ${p}_{i+}=P(X={x}_{i})$ and ${p}_{+j}=P(Y={y}_{j})$ are the marginal distributions of X and Y, respectively. *θ*^2^ is the measure of the magnitude of independence between the two categorical variables *X* and *Y*, with *θ*^2^ = 0 if X and Y are independent and $0\le {\theta }^{2}\le \,\min (m,\,k)-1$, where $m=|X|$ and $k=|Y|,$ respectively^16^.

**Figure 1:**
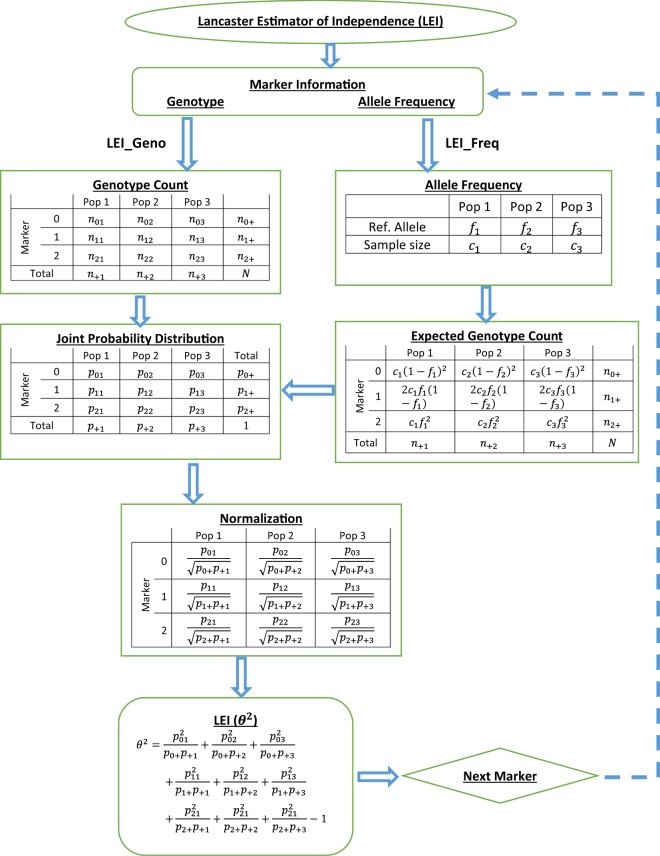
Construction of Lancaster Estimator of Independence (LEI): Analysis flow chart to illustrate LEI_Geno and LEI_Freq analysis scheme.

LEI can use genotype frequency (LEI_Geno) or allele frequency (LEI_Freq) information from SNPs data. Figure 1 shows the flow chart for genotype and allele frequency-based LEI computation algorithm, with the assumption of HWE (random mating, absence of selection and mutation, and infinite population)^23^.

Lancaster Estimator of Independence based on Genotype Data (LEI_Geno). LEI_Geno is based on SNP genotype data. Assume a fixed number of populations where each individual is assigned to one of *k* populations. We take Y as the variable of population membership of the subjects, while X represents a given marker locus coded 0, 1, 2 with respect to the number of reference alleles. Let *n*_*ij*_ (*i* = 0, 1, 2; *j* = 1, 2, …, *k*) be the number of individuals with genotype *i* (*i* = 0, 1, 2) in population *j* (*j* = 1, 2, …, *k*), and $n={\sum }_{j}{\sum }_{i}{n}_{ij}$ be the total sample size. We estimate ${p}_{ij}$ in Equation (1) as ${\hat{p}}_{ij}=\frac{{n}_{ij}}{n}$. Then, LEI_Geno is the estimate of *θ*^2^ as:

$${\hat{\theta }}^{2}={\sum }_{i,j}\frac{{\hat{p}}_{ij}^{2}}{{\hat{p}}_{i+}{\hat{p}}_{+j}}-1.$$

Lancaster Estimator of Independence based on Allele Frequency Data (LEI_Freq). LEI can also be constructed from population allele frequency statistics. It is here referred to as LEI_Freq. For a given marker, let *f*_*j*_ represent the population reference allele frequency in the *j*^*th*^ population with *c*_*j*_ indicating the number of individuals in the *j*^*th*^ population. Then, under the assumption of HWE, the expected genotype counts of genotype 0, 1, and 2 in the *j*^*th*^ population are ${\hat{n}}_{0j}={c}_{j}{(1-{f}_{j})}^{2},{\hat{n}}_{1j}=2{c}_{j}\,{f}_{j}(1-{f}_{j}),$ and ${\hat{n}}_{2j}={c}_{j}\,{f}_{j}^{2}$, respectively. If $n={c}_{1}+{c}_{2}+\ldots +{c}_{k}$ is the sample size, then, as in LEI_Geno, we estimate *p*_*ij*_ in Equation (1) as ${\tilde{p}}_{ij}=\frac{{\hat{n}}_{ij}}{n}$. Then, LEI_Freq is the estimate of *θ*^2^ as:

$${\tilde{\theta }}^{2}={\sum }_{i,j}\frac{{\tilde{p}}_{ij}^{2}}{{\tilde{p}}_{i+}{\tilde{p}}_{+j}}-1.$$

Since $m=|X|=3$ and $k=|Y|$, $0\le {\hat{\theta }}^{2},{\tilde{\theta }}^{2}\le \,min(3,\,k)-1$. Note that, when the sample constitutes three or more populations (i.e., $k\ge 3$), $0\le {\hat{\theta }}^{2},\,{\tilde{\theta }}^{2}\le \,2$.

Using LEI_Geno or LEI_Freq, we determine the distribution of LEI values across *M* genotyped markers. The closer the LEI value to 2, the greater the marker informativeness is. A relatively large LEI value indicates that the marker is highly dependent among the $k$ ancestral populations.

#### Principal Components Analysis (PCA)

PCA reduces the dimensionality of a set of variables by transforming the data to a smaller set of uncorrelated variables, called principal components (PCs). PCs are computed from the eigenvalue decomposition of the data matrix X consisting of N rows and L columns. The first principal component (PC1) contains the highest overall variance, and each of the successive components (PC2, PC3, etc.) contains the highest residual variance. Corresponding to the *i*^*th*^ PC is the *i*^*th*^ eigenvector ${a}_{i}=({a}_{i1},{a}_{i2},\ldots ,{a}_{iL})$. The features with the highest magnitudes of coefficient ${a}_{ij}$ are the most associated features with the variation in the corresponding *i*^*th*^ PC. Thus, the coefficient $|{a}_{ij}|$ can be viewed as the weight associated with the feature *j* corresponding to the *i*^*th*^ PC.

We apply PCA on the data matrix X with N samples (rows) genotyped on L SNPs (columns) coded 0, 1, or 2 based on the reference allele counts. We choose two eigenvectors ${a}_{1}=({a}_{11},{a}_{12},\ldots ,{a}_{1L})$ and ${a}_{2}=({a}_{21},{a}_{22},\ldots ,{a}_{2L})$ that correspond to the first two principal components PC1 and PC2 for the feature selection. Each marker is assigned a score of ${b}_{j}=\,Max(|{a}_{1j}|,\,|{a}_{2j}|)$, and the markers are ranked based on the scores. PCA feature selection method was executed in Python using Scikit-learn^24^.

#### Random Forest (RF)

A random forest classifier is an ensemble of independently constructed classification and regression decision trees (CART) on various bootstrap samples drawn from the input training data, and it uses averages to improve predictive accuracy and control overfitting^25,26^. The method is supervised in the sense that prior knowledge of the classification of the samples is used to train each model in the ensemble. Training a random forest classifier implicitly computes a feature importance measure, called the Gini importance measure, for each feature used in the training data. A larger Gini measure indicates a higher overall discriminative value of the feature for the classification problem^27^. We trained a random forest classifier on the reference genotype data and ranked the markers based on the Gini importance measure. RF was executed in Python with default values to determine the best split with the construction of 500 trees^24^.

#### Support Vector Machine (SVM)

SVM incorporates a weighting method designed to extract and rank features within each SVM class predictor^28,29^. As a supervised learning method, linear SVM trains the classifier by constructing the maximum-margin hyperplane with weight vectors in the data space ${{\mathbb{R}}}^{m}$ resulting in a weight ${\boldsymbol{w}}={\sum }_{{\boldsymbol{i}}}{{\boldsymbol{\alpha }}}_{i}{{\boldsymbol{y}}}_{{\boldsymbol{i}}}{{\boldsymbol{x}}}_{i}$; where α is the vector of Lagrange coefficients, *y*_*i*_ is the response, and *x*_*i*_ is the input vector of *m* features in the data. We extend SVM to a multiclass environment using linear kernel with a *one-versus-the-rest* approach. Using this approach for k distinct populations, we obtain k different weight vectors ${w}_{i}\in {{\mathbb{R}}}^{m},\,i\,=\,1,\,2,\ldots ,\,k$. To construct the feature selection method, we take the average of absolutes, ${{\boldsymbol{\omega }}}_{m}={\sum }_{i}\frac{|{w}_{i}|}{k}$. Each element of *w*_*m*_ is a score for the corresponding marker; the markers are ranked in the decreasing order of the score. The majority of the values will be close to zero and of little importance, whereas the values that will be large tend to discriminate well. SVM was executed using Python with default setting^24^.

### Comparison of feature selection methods: classification accuracy

We implemented a multinomial logistic regression model for the population classification using top-ranked markers selected from LEI, PCA, RF, and SVM. Let k be the number of populations, n = the number of top-ranked markers, and ${p}_{i}=P(Y=i),\,i=1,\,2,\ldots ,k$ be the probability that a given subject belongs to the *i*^*th*^ population, the multinomial logistic regression model is given by:

2 $$log(\frac{{p}_{i}}{{p}_{k}})={\beta }_{0i}+{\beta }_{1i}{X}_{1i}+\ldots +{\beta }_{ni}{X}_{ni},$$

where ${p}_{i}=\frac{{e}^{{\beta }_{0i}+{\beta }_{1i}{X}_{1}+\ldots +{\beta }_{ni}{X}_{n1}}}{1+{e}^{{\beta }_{0i}+{\beta }_{1i}{X}_{1}+\ldots +{\beta }_{ni}{X}_{n1}}}$, ${p}_{k}=\frac{1}{1+{e}^{{\beta }_{0i}+{\beta }_{1i}{X}_{1}+\ldots +{\beta }_{ni}{X}_{n1}}}$ and $i=1,2,\ldots ,k-1$, ${X}_{ji}\in 0,1,2$ is the genotype of the ith individual at the jth marker in the ranked list of n markers. The genotype is coded 0, 1, or 2 based on the count of reference allele.

To compare the feature selection methods (LEI_Geno, LEI_Freq, PCA, RF, and SVM), we assembled the sets ${M}_{r},r=1,\,2,\,3,\ldots ,\,40$, of the top-ranked $r$ markers from each method and fitted the regression model based on the selected markers. The prediction probabilities of each subject to the k populations were computed using the leave-one-out cross-validation (LOOCV) approach. Then, the subjects were classified to one of the *k*^*th*^ population based on the maximum of predicted probability *p*_*i*_ using the model. The confusion matrices for the models were constructed to compute the classification accuracy for each feature selection method. We used the R package caret for fitting the multinomial logistic regression model with LOOCV approach^30^.

We further evaluated the classification performance of the features selection methods using the multinomial version of the area under the receiver operating curve (AUC) statistics^31^. AUC is the probability that a random true positive is ranked higher to be identified as a positive than a true negative^32^. It is a quantitative measure of performance of a classifier and useful in comparing the performance of different classifier. In a two-class classifier problem, a single numerical value will be computed as -

$$AUC=1-0.5(\frac{x}{n}+\frac{e-x}{m})$$

where n = true negatives, m = true positives, *e* = classification errors, and x = false positives^31^. For the multiclass classification problem (3-way population classification in our case), a multinomial version of AUC can be computed. Suppose we have a classification problem with c classes (c ≥ 3). Assuming each class as a true positive class and rest as true negative, AUC_i_ for each class i can be computed as above. Then multinomial AUC (mAUC) of the classifier can be computed as the weighted sum of the AUC_i_ across all classes, i.e.,

3 $$mAUC={\sum }_{i=1}^{c}AU{C}_{i}\times {p}_{i},$$

where the weight ${p}_{i}$ is the proportion of the class i^31^.

### Comparison of feature selection methods for admixture: root mean square error (RMSE)

To evaluate the performance of the LEI against PCA, RF, and SVM, we ran each of these feature selection methods on the reference datasets and the top-ranked marker sets *A*_*r*_ of varying size $r=5,\,10,\ldots ,\,100,\,150,\ldots ,1000$. The subsets were then run using ADMIXTURE with the reference populations for estimating the individual’s admixture proportion $q$^33,34^.

To assess the accuracy of the admixture proportion estimates, the root mean square error (RMSE) was used as defined below for each of the 2-way and 3-way admixture^35^:

4a $$RMS{E}_{2}(Q)=\sqrt{\frac{1}{n}{\sum }_{i=1}^{n}{({\hat{q}}_{i}-{q}_{i})}^{2}}\,(\mathrm{two} \mbox{-} \mathrm{way}\,{\rm{admixture}}),$$

4b $$RMS{E}_{3}(Q)=\sqrt{\frac{1}{nK}{{\rm{\Sigma }}}_{i}{{\rm{\Sigma }}}_{k}{({\hat{q}}_{ik}-{q}_{ik})}^{2}}\,(\mathrm{three} \mbox{-} \mathrm{way}\,{\rm{admixture}})$$

where *n* is the number of individuals in the sample, K is the number of ancestral populations, *q*_*i*_ (and *q*_*ik*_) represents the true ancestry proportion (known simulated ancestry), and ${\hat{q}}_{i}$(and ${\hat{q}}_{ik}$) defines the estimated ancestry proportion for each subject *i*. ADMIXTURE software was used to analyze the data^33^.

## Results

### Distribution of LEI

**Figure 2:**
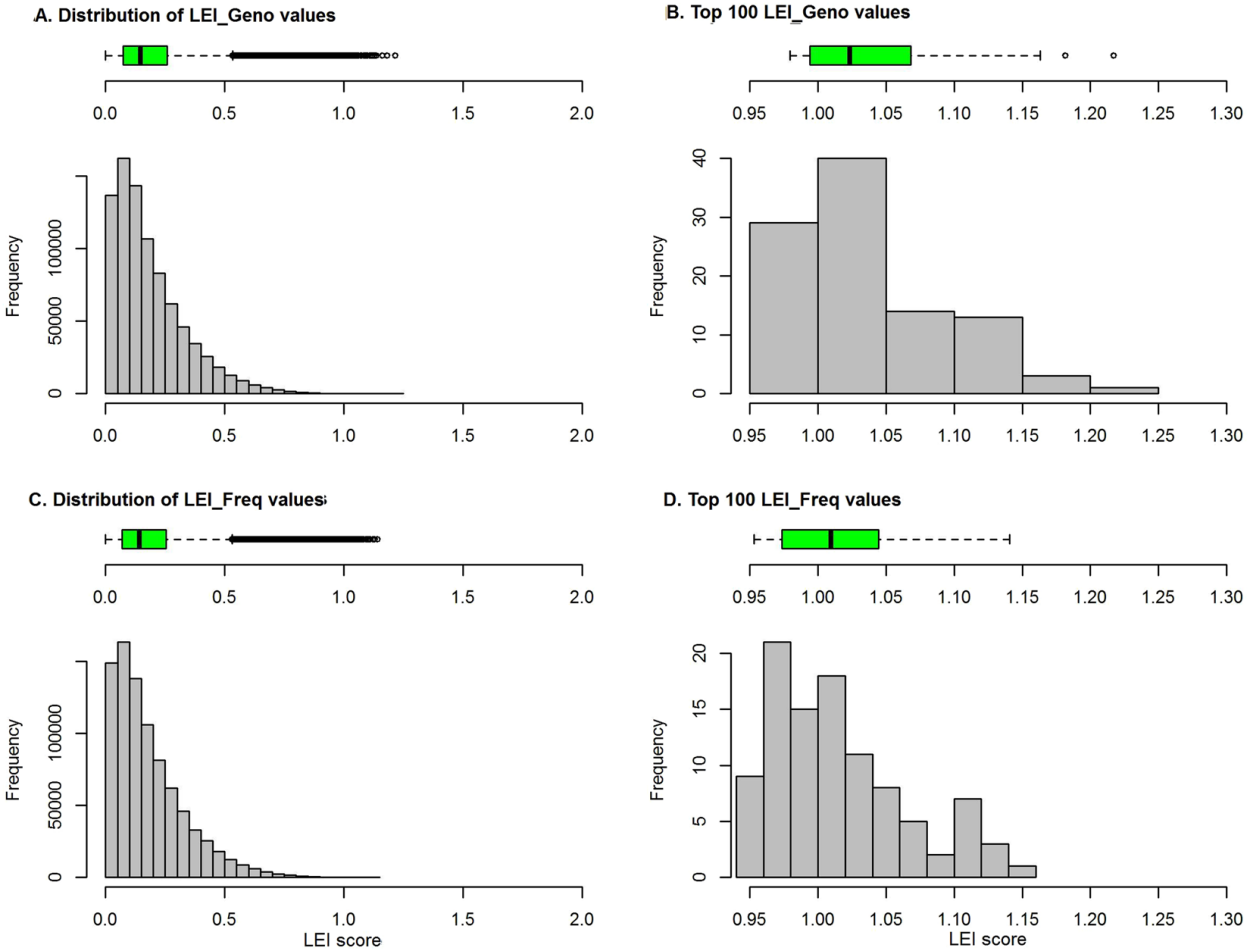
Distribution of LEI values. Distribution of Lancaster Estimator of Independence (LEI) across 857112 SNPs from 1000 Genomes Phase 3 with a total of 310 subjects belonging to the following populations: CEU (Utah residents with Northern and Western European ancestry: n = 99), CHB (Han Chinese in Beijing: n = 103), and YRI (Yoruba in Ibadan, Nigeria: n = 108). (**A**) Distribution of LEI scores based on the genotype data (LEI_Geno). (**B**) Distribution of the top 100 LEI_Geno scores. (**C**) Distribution of LEI scores based on the allele frequency data (LEI_Freq). (**D**) Distribution of top 100 LEI_Freq scores.

We first assembled the LEI values based on genotype and allele frequency data (LEI_Geno and LEI_Freq). For the three populations of CEU, YRI, and CHB, the resulting distributions for both LEI_Geno and LEI_Freq are similarly skewed to the right (Fig. 2A,C). The distribution of the top 100 LEI scores (Fig. 2B,D) shows that the top scores from LEI_Geno are skewed to the right further from LEI_Freq. Over 90% of the LEI values fall near zero, indicating independence between markers and populations; thus, the majority of the markers are not informative for ancestry. The maximum score for LEI_Geno (maximum = 1.217) is slightly greater than that for LEI_Freq (maximum = 1.141). The median and other quartiles of LEI_Geno are also slightly greater than those of LEI_Freq (LEI_Geno: median = 0.1447, first quartile = 0.074, third quartile = 0.2573; LEI_Freq: median = 0.1418, first quartile = 0.070, third quartile = 0.255). For both LEI_Geno and LEI_Freq, SNPs with value greater than 0.532 appeared to be outliers and can be regarded as SNPs with the highest discriminative power among the three continental populations (Fig. 2A,B).

The two LEI measures (i.e., LEI_Geno and LEI_Freq) are highly correlated with Pearson’s correlation coefficient (r = 0.9955). The top-ranked markers overlap between the two methods. Seventy-three markers among the top-ranked 100 SNPs from LEI_Geno and LEI_Freq are common. Similarly, 793 among the top-ranked 1000 markers from LEI_Geno and LEI_Freq are overlapping markers. In addition, two SNPs (rs1325421 and rs11085023) among the top 100 LEI_Geno SNPs show the strongest discriminative signal among populations (Fig. 2B).

### Informative markers selection and overlap analysis

For LEI_Geno and LEI_Freq, we ranked the markers based on the respective scores. In PCA, we used the first two PCs for feature selection and ranked the markers based on the maximum magnitude of the coefficients of the corresponding first and second eigenvectors (see the Method section). The first PC accounted for 13.6% of the variation in the data, the second PC for 6.4%; and thereafter, each PC accounted for less than 1%. For SVM, we ranked each marker using the absolute average weight *w*_*m*_ for each marker. For RF classifier, we ranked the markers based on the Gini importance measure.

**Figure 3:**
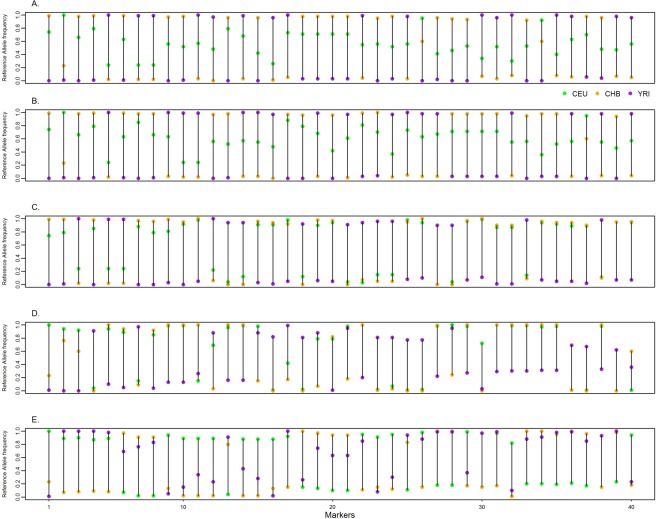
Top ranked markers allele frequency variation among feature selection methods. The top 40 markers from each feature selection were ordered by allele frequency difference for each of the three reference populations: CEU (Utah residents with Northern and Western European ancestry from the CEPH collection), CHB (Han Chinese in Beijing, China), and YRI (Yoruba in Ibadan, Nigeria). (**A**) Lancaster Estimator of Independence based on genotype data. (**B**) Lancaster Estimator of Independence based on population allele frequencies. (**C**) Principal component analysis. (**D**) Random forest (**E**) Support vector machine.

Figure 3 shows the difference in allele frequencies of 40 top-ranked SNPs selected from each method, ordered by the maximum of the pairwise allele frequency differences among the three populations CEU, YRI, and CHB. These markers showed large allele frequency differences among the three populations. The maximum of the pairwise allele frequency differences for LEI_Freq range from 0.94 to 0.99 with a mean of 0.96, whereas for LEI_Geno, they range from 0.91 and 0.99 with a mean of 0.95. The PCA, RF, and SVM all ranked SNPs with relatively smaller allele frequency differences, with the narrowest differences found in the RF markers whose average maximum is 0.794, followed by SVM with mean 0.846, and PCA with mean frequency differences 0.923. For RF, the allele frequency differences range between 0.59 and 0.99 while for SVM, the differences are between 0.76 and 0.99.

### Comparison of feature selection methods for continental ancestry

**Figure 4:**
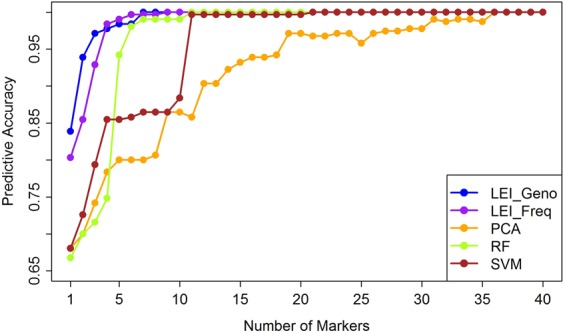
Comparison of predictive accuracy estimates for continental ancestry. Multinomial logistic regression models –based on top-ranked 40 SNPs using Lancaster Estimator of Independence constructed from genotype subject data (LEI_Geno), constructed from SNP/Population summary statistics (LEI_freq), Principal Components Analysis (PCA), Random Forest (RF), and Support Vector Machine (SVM) feature selection methods.

For each feature selection method, we select top-ranked markers n = 1, 2, …, 40 and fit the multinomial logistic regression model to build predictive classification models with leave-one-out cross-validation (LOOCV). The subjects are classified to one of the three populations based on the predicted probability *p*_*i*_ using the fitted model and the classification accuracy is assessed. The singleton top ranked marker from LEI_Geno produced the highest classification accuracy (0.84) followed by LEI_Freq (0.80), SVM (0.68), PCA (0.68) and RF (0.67) (Fig. 4). The prediction accuracy for both LEI_Geno- and LEI_Freq-derived markers reached ~97% when four top ranked markers were used. Six markers from RF approach achieved similar accuracy while 11 and 31 markers were required to achieve similar accuracy under SVM and PCA-based selection approaches (Fig. 4). As expected, the classification accuracy increases with the number of markers. Seven and nine markers selected respectively from LEI_Geno and LEI_Freq produced 100% classification rate whereas 11 markers selected from RF, 21 markers from SVM, and 36 markers from PCA produced similar accuracy. The 95% confidence interval of accuracy rate showed no significant differences in the accuracy rate between the two LEI-based approaches but showed significantly better classification accuracy than that from PCA and SVM based markers (Supplementary Table 1). The difference in accuracy rate between RF and LEI based classifiers was significant when less than 6 markers were used but insignificant for 6 or more markers. Similarly, mAUC (multinomial AUC) value of the classifiers with n = 1, 2, …, 10 top features from each method support similar insights that when fewer markers were used LEI-based markers performed better than other methods. The results show that the performance of LEI_Geno, LEI_Freq, RF, and SVM are similar, whereas PCA method requires more markers to achieve comparable classification accuracy.

**Figure 5:**
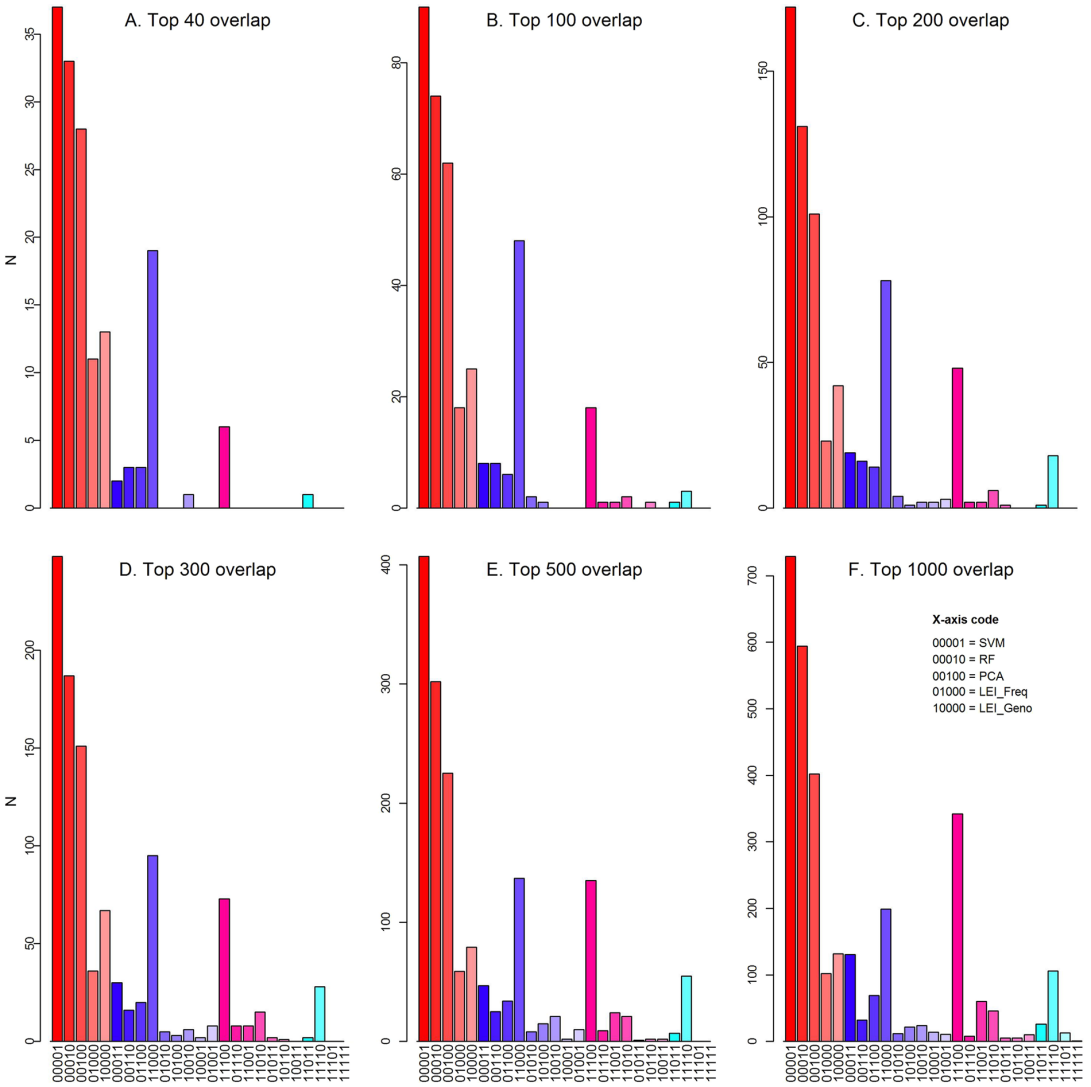
Overlap of top ranked markers selected by different feature selection methods. (**A**) N = 40, (**B**) N = 100, (**C**) N = 200, (**D**) N = 300, (**E**) N = 500, (**F**) N = 1000. A 5-digit binary vector was assigned to each marker, where each digit represents one of the five feature selection methods, with the first digit indicating the marker was selected (or not) by Lancaster Estimator of Independence constructed from genotype subject data (LEI_Geno), followed by LEI constructed from SNP/Population summary statistics (LEI_freq), PCA, RF, and SVM. The 1 digit indicates the SNP was selected by method as one of the top N markers. Bars are clustered based on the total number of methods represented by the bar with varying color gradients (red color bars = bars correspond to the five individual methods, blue color bars = bars correspond to a pair of methods, and so on).

Figure 5 shows the overlap analysis of top-ranked markers separating the CEU, YRI, and CHB continental populations among the five methods. The list of top 1000 markers from each method are provided in Supplementary Table 2. Among the top 40 markers, LEI_Geno and LEI_Freq shared the largest overlap between any two methods with 26 shared markers, of which 19 were shared only between the two methods. There were 6 markers shared among LEI_Geno, LEI_Freq, and PCA (Fig. 5A) and one marker was shared among LEI_Geno, LEI_Freq, RF, and SVM. From the top-ranked 40 markers selected by each method, no marker was found common in all methods. The top-ranked SNP rs1325421 (selected by LEI) has been reported among the highly informative marker for multiple ancestry^36^. We further checked for SNPs in high LD (r^2^ >0.8) with rs1325421 in CEU, YRI, and CHB populations using the 1000 Genomes project reference panel. No SNPs with rs1325421 (within 500 kb region) were in LD among the top 1000 SNPs from PCA, RF, and SVM. This shows that the LEI can pick SNPs with the highest discriminative power among multiple populations. One SNP (rs2675345) was shared among LEI_Geno, LEI_Freq, RF, and SVM. This SNP has been cited in multiple studies related to skin color^37,38^. However, rs2675345 or its LD (r^2^ >0.8) SNPs were not selected by PCA. The overlap analysis conducted for the top 1000 markers show an overall trend where SVM selects the largest number of unique markers, followed by RF and PCA. LEI_Geno and LEI_Freq consistently selected the most overlapping top ranked marker sets (793 from the 1000 markers). From the top 1000 markers, only one marker rs3935973 was overlapped among all five feature selection approaches.

### Comparison of feature selection methods for admixture proportion

#### Root mean square error (RMSE) in admixed ancestry estimation

**Figure 6:**
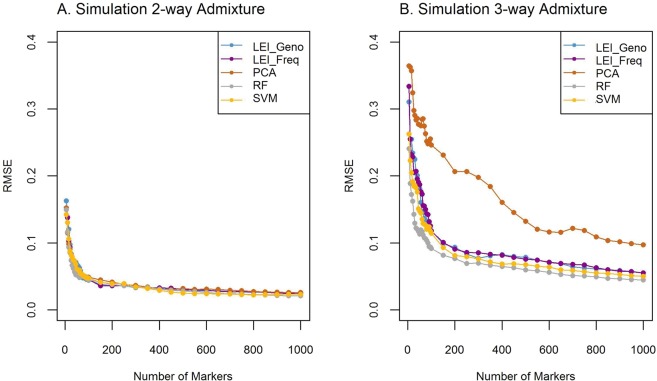
Root mean square error (RMSE) for 2-way and 3-way admixture simulation. Comparisons of ancestry estimation using the top-ranked markers. Lancaster Estimator of Independence constructed from genotype subject data (LEI_Geno), LEI constructed from SNP/Population summary statistics (LEI_Freq), Principal Components Analysis (PCA), Random Forest (RF), and Support Vector Machine (SVM) feature selection methods. Ancestry proportions are estimated using ADMIXTURE for sets of top-ranked markers from each feature selection method. (**A**) Two-way admixture RMSE, 100 simulated individuals from reference populations: CEU (Utah residents with Northern and Western European ancestry from the CEPH collection), and YRI (Yoruba in Ibadan, Nigeria) using true proxy (n = 20,085 SNPs). (**B**) Three-way admixture RMSE, 100 simulated individuals from reference populations: CEU (Utah residents with Northern and Western European ancestry from the CEPH collection), YRI (Yoruba in Ibadan, Nigeria), CHD (Chinese in Metropolitan Denver, Colorado) using true proxy (n = 19,982 SNPs).

RMSE was used to measure the variation (or accuracy) from estimated individual admixture and true ancestry^35^. The RSME for two-way and three-way admixture analyses were computed using (4a) and (4b), respectively. We obtained estimates of individual ancestry proportion (q_i_ estimates) for the admixed groups by running ADMIXTURE using different sets of top-ranked marker genotype data from each of the feature selection methods^33^. Ancestry proportion estimates were obtained by running ADMIXTURE with all the SNPs as the true proxy. Figure 6 shows the RMSE of ancestry proportion from two-way and three-way admixture analyses. The RMSE for two-way analysis is less than 0.05 with the top-ranked 85 markers and results are comparable among all five methods (Fig. 6A). The RMSE stayed almost constant when the number of markers reached 1000. The RMSE is higher for three-way admixture analysis, and the error is higher for PCA-based markers while the other four approaches produce similar results. When the number of markers reached 1000, the RMSE for all methods stabilize around RMSE = 0.09 for PCA and 0.05 for the other four approaches (Fig. 6B). Note that all five methods use the reference populations simultaneously to rank the ancestry informative variants and do not rely on pairwise population comparison. Our results showed that markers based on PCA produce the least accurate ancestry estimates in the three-way admixture while all five methods produce comparable results for two-way admixture.

#### Ancestry proportion estimates based on top-ranked markers

**Figure 7:**
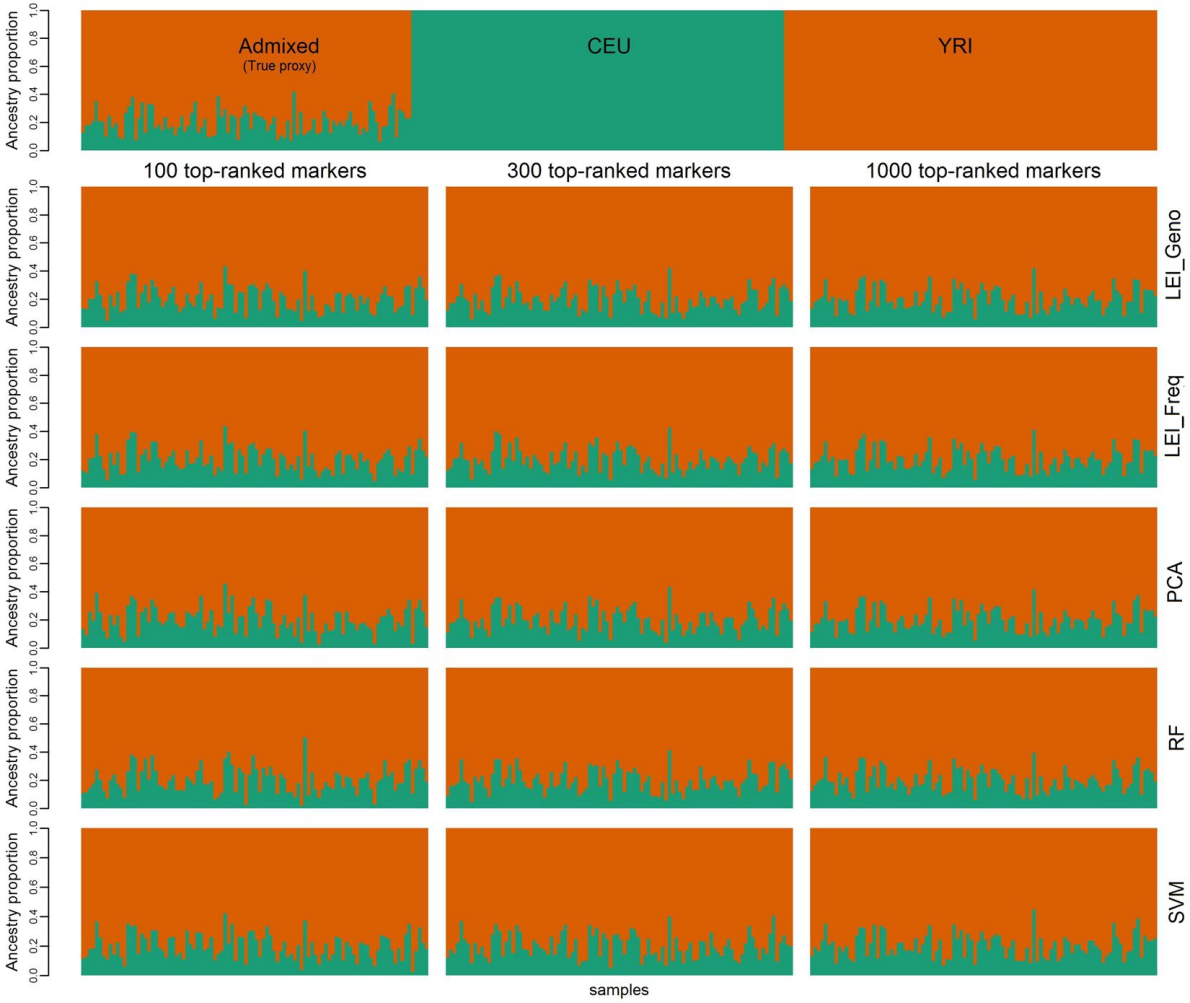
Graphical output for two-way admixture analysis. Inferred ancestry estimates for the 100 simulated admixed individuals using ADMIXTURE. Two references population CEU (Utah residents with Northern create simulate and Western European ancestry from the CEPH collection), and YRI (Yoruba in Ibadan, Nigeria) were used to simulate 2-way admixed population. Each individual is represented by a vertical line. Top most plot represents true proxy (n = 20,085 SNPs) plot for admixed individuals. Each subsquent row represents the plots for 100, 300, and 1000 top-ranked markers coming from Lancaster Estimator of Independence constructed from genotype subject data (LEI_Geno), followed by LEI constructed from SNP/Population summary statistics (LEI_Freq), Principal Components Analysis (PCA), Random Forest (RF), and Support Vector Machine (SVM) feature selection methods.

**Figure 8:**
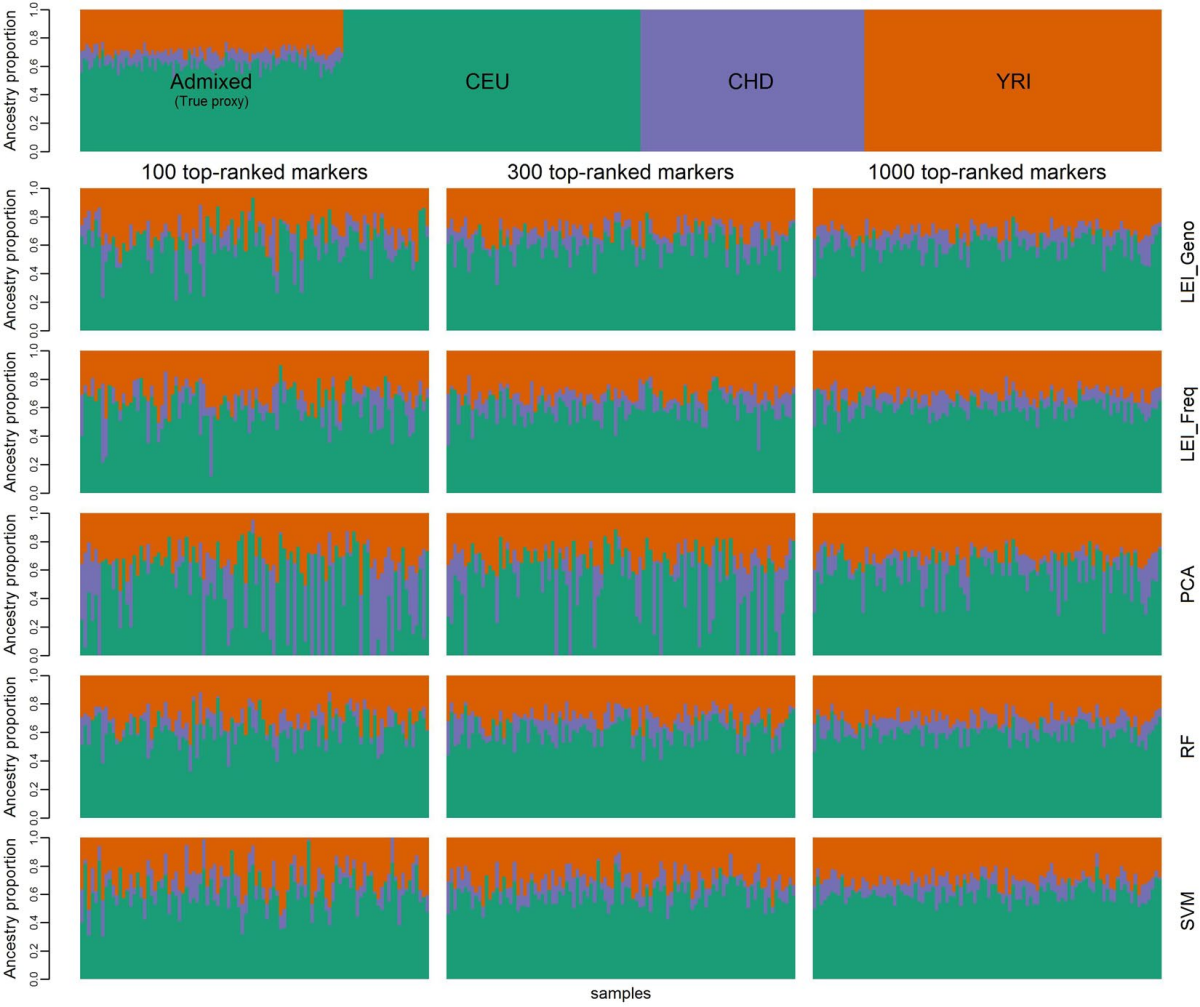
Graphical output for three-way admixture analysis. Inferred ancestry estimates of the 100 simulated admixed individuals using ADMIXTURE. Three reference populations, CEU (Utah residents with Northern and Western European ancestry from the CEPH collection), CHD (Chinese in Metropolitan Denver, Colorado), and YRI (Yoruba in Ibadan, Nigeria) were used to simulate 3-way admixed samples. Each individual is represented by a vertical line. Simulation was based on true proxy (n = 19,982 SNPs). Top most plot represents estimates using the true proxy SNPs for the admixed individuals, followed by the CEU (n = 113), CHD (n = 85) and the YRI (n = 113) reference individuals. Each subsequent row represents the q-plots for 100, 300, and 1000 top-ranked markers coming from Lancaster Estimator of Independence constructed from genotype subject data (LEI_Geno), followed by LEI constructed from SNP/Population summary statistics (LEI_Freq), Principal Components Analysis (PCA), Random Forest (RF), and Support Vector Machine (SVM) feature selection methods.

Figure 7 shows the estimates of ancestry proportions of samples based on 100, 300, and 1000 top-ranked SNPs for two-way simulation. The true proxy estimates were obtained using all markers. As the number of markers increases, slight differences are observed among the methods. Figure 8 shows the estimates of ancestry proportions based on 100, 300, and 1000 top-ranked SNPs for three-way simulation, with the top-panel representing the true proxy of the ancestry proportion. In 3-way admixture, the distinctions between the methods are evident, even if the number of markers increases. The ancestry proportion estimates are similar among the LEI_Geno, LEI_Freq, RF, and SVM, but they differ markedly from the PCA estimates.

**Figure 9:**
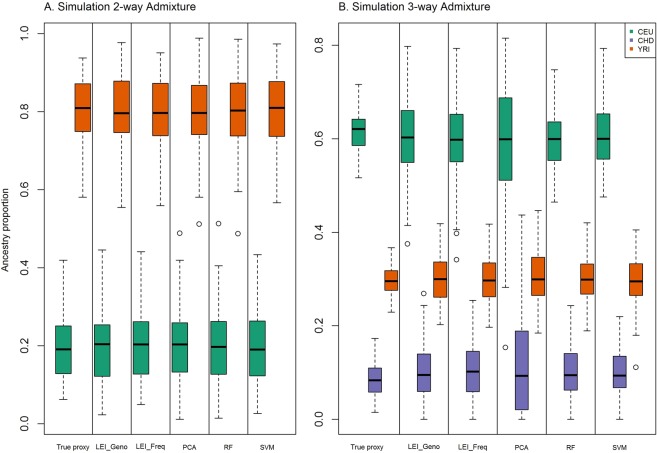
Ancestry proportions for 2-way and 3-way admixture simulation. Comparisons of ancestry estimation using true proxy (All) and top-ranked markers for Lancaster Estimator of Independence constructed from genotype subject data (LEI_Geno), LEI constructed from SNP/Population summary statistics (LEI_Freq), Principal Components Analysis (PCA), Random Forest (RF), and Support Vector Machine (SVM) feature selection methods. Ancestry proportions are estimated using ADMIXTURE. (**A**) Two-way admixture boxplots, 100 simulated individuals from reference populations: CEU (Utah residents with Northern and Western European ancestry from the CEPH collection), and YRI (Yoruba in Ibadan, Nigeria). Ancestry estimates using true proxy (n = 20,085 SNPs) and 85 top-ranked SNPs from each feature selection method. (**B**) Three-way admixture boxplots, 100 simulated individuals from reference populations: CEU (Utah residents with Northern and Western European ancestry from the CEPH collection), YRI (Yoruba in Ibadan, Nigeria), CHD (Chinese in Metropolitan Denver, Colorado). Ancestry estimates using true proxy (n = 19,982 SNPs) and 1000 top-ranked SNPs from each feature selection method.

Using the simulated data, we also compared the distribution of the estimated ancestry proportions for two-way and three-way admixture (Fig. 9). The distributions of the estimates based on the top 85 informative markers in 2-way simulation demonstrate that all the feature selection methods performed comparably well with the true proxy in 2-way admixture analysis. The distributions of the estimates based on the top 1000 informative markers for the three-way simulation show a larger variation of estimated ancestry proportions among all five methods in comparison to those from the true proxy estimates (Fig. 9B). The ancestry proportion estimates based on RF and SVM methods were appeared to perform marginally better than LEI-based approaches. However, both RF and SVM were based on the individual level genotype data whereas LIE_Freq was based on the summary level data. Note that, in two-way admixture, eighty-five markers were required for each feature selection method for the RMSE to fall below 0.05 (Fig. 6A); in contrast, for 3-way admixture analysis, 1000 markers were required for the RMSE to fall below 0.05 except for PCA (Fig. 6B). Figure 6B showed the RMSE for PCA was higher (RMSE = 0.09) as compared to the other feature selection methods. The difference in performance of the RMSE for two-way (Fig. 6A) vs. three-way (Fig. 6B) admixture analysis could be due to noise in estimating ancestry variations contributed from two vs. three sources of ancestral population.

We further compared the absolute difference of the African ancestry proportion between the estimates using the selected top-ranked markers and the estimates using all the markers (true proxy). In two-way analysis, the number of individuals within the difference of 0.05 from the true proxy ranged from 65 for LEI_Freq to 66 for RF with LEI_Geno capturing 71 (Table 1A). Similarly, for three-way admixture, the numbers of individuals within the difference of 0.05 from the true proxy were 54, 59, 28, 60, and 69 for LEI_Geno, LEI_Freq, PCA, RF, and SVM, respectively (Table 1B). Further, Table 1 showed the 95% confidence intervals for the African ancestry proportion estimated from the five methods for both two-way and three-way admixtures. The confidence intervals for each method contained the mean true proxy estimate.

**Table 1:** African ancestry proportion comparison in 2-way and 3-way admixture simulation scenario.

Feature selection method	Range (African ancestry proportion)	Mean (average African ancestry proportion)	95% CI (95% confidence interval)	^#^Subjects within difference 0.05 (estimated vs true proxy ancestry)
**A. Simulated two-way Admixture**				
True proxy	[0.5806, 0.9376]	0.7997	[0.7829, 0.8165]	NA
LEI_Geno	[0.5546, 0.9773]	0.7994	[0.7813, 0.8175]	71
LEI_Freq	[0.5594, 0.9509]	0.7986	[0.7808, 0.8164]	65
PCA	[0.5118, 0.9888]	0.7987	[0.7805, 0.8169]	66
RF	[0.4872, 0.9858]	0.7990	[0.7798, 0.8182]	66
SVM	[0.5665, 0.9737]	0.7997	[0.7820,0.8174]	72
**B. Simulated three-way Admixture**				
True proxy	[0.5165, 0.7163]	0.6164	[0.6079, 0.6248]	NA
LEI_Geno	[0.3753, 0.7975]	0.5972	[0.5809, 0.6135]	54
LEI_Freq	[0.3413, 0.7937]	0.5949	[0.5786, 0.6112]	59
PCA	[0.1537, 0.8154]	0.5820	[0.5544, 0.6096]	28
RF	[0.4647, 0.7478]	0.5982	[0.5858, 0.6106]	60
SVM	[0.4758, 0.7934]	0.6062	[0.5934, 0.6190]	69

African Ancestry proportions estimated using ADMIXTURE. (A) Simulated two-way admixture. (B) Simulated three-way Admixture. For both two-way and three-way admixture analysis, 100 subjects were simulated using markers common to the reference populations on chromosome 22 from HapMap III. Two-way admixed subjects were simulated from reference populations CEU and YRI and three-way were simulated from CEU, CHD, and YRI. African ancestry proportion estimates for two-way and three-way admixture were based on 85 top-ranked SNPs and 1000 top-ranked markers, respectively and from each feature selection method: Lancaster Estimator of Independence constructed from genotype subject data (LEI_Geno), LEI constructed from SNP/Population summary statistics (LEI_Freq), Principal Components Analysis (PCA), Random Forest (RF), and Support Vector Machine (SVM) feature selection methods. Columns: Feature selection method, Range = range of African ancestry proportion estimates for 100 subjects, Mean = average African ancestry proportion estimate for 100 subjects, 95% CI = 95% confidence interval, ^#^Subjects within difference 0.05 = number of subjects with the absolute value of the difference between the estimated ancestry proportion and the true proxy estimate is less than or equal to 0.05; NA = not applicable. True proxy are the admixture proportion estimates based on all the markers used in each simulation. CEU (Utah residents with Northern and Western European ancestry from the CEPH collection), YRI (Yoruba in Ibadan, Nigeria), CHD (Chinese in Metropolitan Denver, Colorado).

#### Correlations among feature selection methods in admixed samples

**Figure 10:**
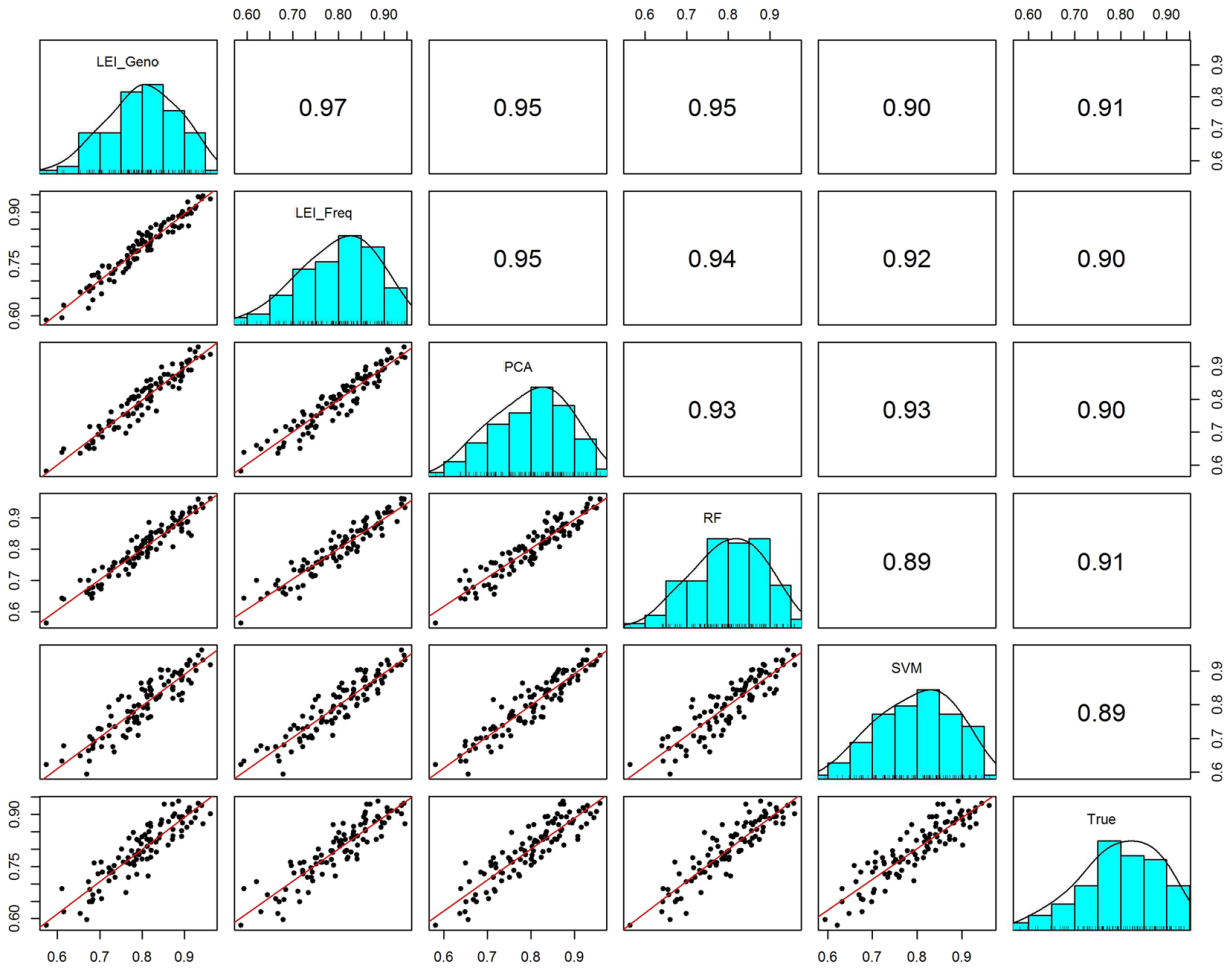
Correlation matrix plot comparisons between feature selection method estimates for simulated 2-way admixed population. Numbers indicate the correlation coefficients and the lines indicate linear fitting for the 100 simulated data point from two reference populations: CEU (Utah residents with Northern and Western European ancestry from the CEPH collection), and YRI (Yoruba in Ibadan, Nigeria). The correlation plots are constructed for individual estimates of African ancestry proportion from ADMIXTURE using 85 top-ranked SNPs based on Lancaster Estimator of Independence constructed from genotype subject data (LEI_Geno), Lancaster Estimator of Independence constructed from the population allele frequency data (LEI_Freq), Principal Components Analysis (PCA), Random Forest (RF), Support Vector Machine (SVM) as well as the true proxy estimates based on n = 20,085 SNPs.

We compared estimates of individual African ancestry for both two-way and three-way simulated samples. Figure 10 shows the pairwise correlations matrix between the feature selection methods for simulated two-way admixed population and the scatter plot (true vs. the estimates) on each feature selection method. The estimates of African ancestry proportion from the different feature selections were highly correlated with correlation ranging from r = 0.888 between the RF and SVM to r = 0.9695 between LEI_Geno and LEI_Freq (Fig. 10). The two-way admixture correlation for each method against the estimates based on the true proxy ranged from r = 0.8927 for SVM to r = 0.9066 for RF, and r = 0.9042 for LEI_Freq (Fig. 10). The ancestry estimates from LEI_Freq were highly correlated with the estimates from LEI_Geno with r = 0.9695.

**Figure 11:**
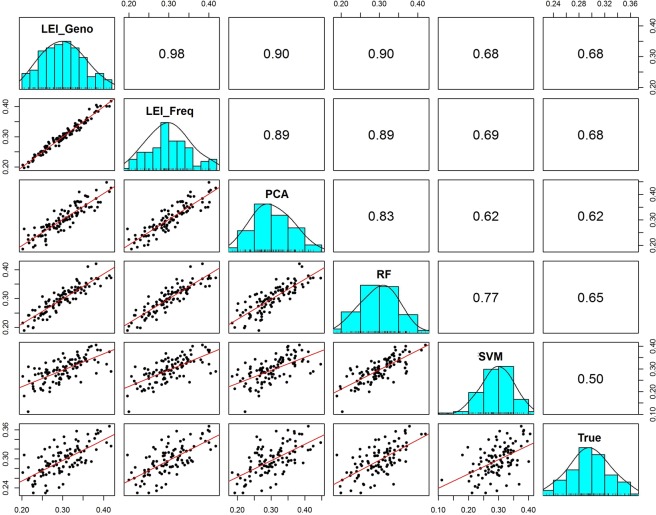
Correlation matrix plot comparisons between feature selection method estimates for simulated 3-way admixed population. Pearson correlation and scatter plots of 100 simulated individuals from three reference populations: CEU (Utah residents with Northern and Western European ancestry from the CEPH collection), CHD (Chinese in Metropolitan Denver, Colorado), and YRI (Yoruba in Ibadan, Nigeria). The correlation plots are constructed for individual estimates of African ancestry proportion from ADMIXTURE are using 1000 top-ranking SNPs and feature selection methods Lancaster Estimator of Independence constructed from genotype subject data (LEI_Geno), Lancaster Estimator of Independence constructed from population allele frequency data (LEI_Freq), Principal Components Analysis (PCA), Random Forest (RF), Support Vector Machine (SVM) as well as the true proxy estimates based on n = 19,982 SNPs.

For three-way admixture analysis, we focused on the estimates of African ancestry proportions. Figure 11 shows the pairwise correlations matrix between the feature selection methods for simulated three-way admixed population and the scatter plot (true vs. the estimates) on each feature selection method. The scatter plots consist of true vs. the estimates based on each feature selection method (Fig. 11). Among the pairwise comparison, the ancestry estimates based on PCA and SVM were the least correlated with r = 0.6169, and the estimates from LEI_Geno and LEI_Freq were the most correlated with r = 0.9834. The correlation for each method against the estimates based on all the markers (true proxy estimates) ranged from r = 0.5048 for SVM to r = 0.6847 for LEI_Freq. The three-way admixture correlation between the estimates based on LEI_Geno and LEI_Freq was r = 0.9834. The LEI-based approaches were the most correlated with the true proxy (r = 0.6826 for LEI_Geno and r = 0.6847 for LEI_Freq) while SVM was the least correlated (r = 0.5048). Figure 11 further showed that SVM was the least correlated with the other four methods.

## Discussion

The main objective of this study was to develop an efficient algorithm that can select the most informative markers to infer multi-level ancestry proportions in admixed populations from summary-level allele frequency data without using the entire individual-level genotype data. We presented Lancaster Estimator of Independence (LEI) as a novel feature selection algorithm in multi-ancestry populations.

The availability of fast and efficient algorithms for ancestry inference and admixture proportion facilitates the analysis of big omics data. However, the selection of informative markers that are useful in assigning individuals to the correct continental origins or in estimating the ancestry proportions in admixed samples remains a computational challenge. In this study, we compared the performance of LEI in selecting informative markers with existing machine learning approaches including PCA, RF, and SVM using real and simulated data in a logistic regression framework. The number of informative markers required to build an efficient classifier was found to be similar among these methods with the exception of PCA, which requires a larger number of markers to attain a comparable accuracy (Fig. 4). Using 2-way admixed simulated data, we further showed that markers selected for ancestry proportion estimation based on LEI_Freq performed equally to RF and SVM and better than the PCA (Fig. 6A). From the three-way admixed simulation data, we found that RF-based markers resulted in the best RSME followed by SVM while both LEI_Geno and LEI_Freq performed marginally similar to the RF and SVM (Fig. 6B). In random forest, the top-ranked SNPs contribute the most independent information in prediction of the population classes and hence ancestry inference in three-way admixture. However, the machine-learning approaches including RF, PCA, and SVM require individual-level genotype data which can be computationally expensive when applied to the whole genome-wide genotyped data. For example, training SVM is computationally costly as it depends on the number of input variables, especially when applying to human genotype data^39^. In contrast, one of the advantages of LEI is that it can be applied to the population-level allele frequency data instead of the individual-level genotype data and provides a more efficient alternative to the existing computationally- and time-consuming genotype-based AIMs selection for multi-ancestry inference. Population-level allele frequency data are more convenient to store, share, and compute and are often more readily available than the individual-level genotype data. In random forest, subsets of features are randomly selected to build decision trees, and the feature importance measure (the Gini impurity index) changes between the different runs of RF. Also, the Gini impurity index depends on the number of trees assembled in the RF. Therefore, assigning a unique ranking score for each feature using RF may not be appropriate. PCA is a computationally efficient approach resulting low-dimensional principal components that cluster individuals based on ancestry without specifying the number of ancestral populations in the study. However, in PCA, the choice of the number of PCs is always subjective, and there is no unique way to assign a ranking score that accounts for all the PCs even when the same number of PCs is used^40^. In contrast, our likelihood-based method, LEI, explicitly focuses on summary level allele frequencies without requiring individual level genotype data. LEI provides an efficient alternative to the existing computationally and time-consuming genotype-based analysis for ancestry inference.

Our results show that by applying LEI_Freq build upon the population allele frequencies, the performance is not compromised at both the continental population classification and ancestry proportion inference in admixed populations. In addition, the LEI method could potentially be applied to develop ancestry informative markers for admixed samples with more than two ancestral populations such as Latino/Hispanic (work in progress). Existing approaches of selecting AIMs in three-way admixed populations utilize the pairwise measures of marker informativeness and aggregate the pairwise informative markers into a single set of ancestry informative markers^14^. The Wright’s fixation index (F_ST_), although commonly used as a pairwise measure of marker informativeness, can be applied for selecting AIMs from multiple ancestral population using the population allele frequencies (Supplementary Text S1)^41–43^. We have compared the performance of top ranked markers selected based on the global F_ST_ with LEI, PCA, RF and SVM using CEU, CHB, and YRI ancestral populations (Supplementary Table 1). The classification accuracy rate of the global F_ST_ based the top-ranked marker was 0.6806, which was similar to the results obtained from SVM and PCA, but lower than the LEI based approaches (LEI_Freq: 0.8032). As we increase the informative SNPs to 10, the global F_ST_ based markers still yield lower prediction accuracy (0.8065, 95% CI: 0.758–0.8489) than other methods (including LEI, SVM) but comparable to the PCA-based approach (0.8645; 95% CI: 0.8213–0.9006; Supplementary Table 1). The prediction accuracy for both PCA and F_ST_ reached 0.98 when the top 30 informative markers were used.

In critically evaluating our method development and comparison, it is important to note that our analysis, results and hence interpretations have some important limitations. The performance of the feature selection approaches is tightly linked with the heuristic function used in the feature selections. For instances, PCA loadings from the first two principal components is largely an empirical approach and may not be the optimal approach to select features. We compare LEI with the most commonly used machine learning approaches, and comparison with other specialized machine learning approaches such as sparse PCA^44^ and sparse SVM^45^ warrant further evaluation. We have developed and compared the top informative markers from the different approaches and no LD filtering was applied in the selection process. Consequently, a better subset of independent AIMs may exist. Selecting AIMs is a two-step process: First, assign a measure of marker informativeness for all the available markers using measures of marker informativeness and appropriate reference panels, and second, select top informative markers after appropriate filtering criteria such as pruning based on the LD or genetic distance for the study population. Additionally, our results are based on biallelic SNPs allele frequencies which are the most common type in human genome. However, the LEI approach is generalizable to infer population structure from multi-allelic markers. Nevertheless, the ability of LEI to develop informative feature selection panel and determine ancestry proportion in multi-ancestry admixed populations by leveraging allele frequency data without requiring the individual level genotype data from ever-increasing genomic resources is appealing to most biologists with limited computer programming background.

In summary, we developed an allele frequency-based LEI method to identify smaller subsets of SNPs for population classification and ancestry analysis in admixed populations. LEI is an efficient feature selection method that has the potential to select informative markers in continental populations as well as multi-ancestry admixed populations from allele frequency data. Building LEI based on allele frequency data without requiring the individual-level genotype data is appealing in the big data era.

## Supplementary information

Supplementary Text S1

Supplementary Table 1

Supplementary Table 2

## Data Availability

*LEI* is implemented in the R programming language and R-source code is freely available to download and included in the Appendix. The R-codes can also be assessed freely from the github page https://github.com/MershaLab/LEI.
