# Supplementary material for: LEI: A Novel Allele Frequency-Based Feature Selection Method for Multi-ancestry Admixed Populations
Source: Sci Rep. 2019 Jul 31;9:11103. doi: 10.1038/s41598-019-47012-y (PMC6668412; doi:10.1038/s41598-019-47012-y)
Supplement: Supplementary file 1 — Supplementary Text S1 [file 41598_2019_47012_MOESM1_ESM.docx]

**LEI: A Novel Allele Frequency-Based Feature Section Method for Multi-ancestry Admixed Populations**

[Michael J. Wathen](mailto:Michael.Wathen@cchmc.org)^1,2#^, Yadu Gautam^2#^, Sudhir Ghandikota^2^, Marepalli B. Rao^1^, and Tesfaye B. Mersha^2*^

^1,2^Division of Biostatistics and Epidemiology, University of Cincinnati, Cincinnati, OH 45229, USA

^2^Department of Pediatrics, Cincinnati Children’s Hospital Medical Center, Cincinnati, OH 45229, USA.

***Corresponding author:**

Tesfaye B. Mersha, Ph.D.
Associate Professor

Cincinnati Children's Hospital Medical Center
Department of Pediatrics
University of Cincinnati

3333 Burnet Avenue

MLC 7037

Cincinnati, OH 45229-3026
Phone: (513) 803-2766

Fax: (513) 636-1657

Email: [tesfaye.mersha@cchmc.org](mailto:tesfaye.mersha@cchmc.org\)

# = Equal Contribution

**Supplementary Text S1**

1. R code for LEI_Geno function

lei <- function(x,y){

temp <- as.matrix(table(x,y))

tbl <-as.matrix(prop.table(temp))

nrows <- nrow(tbl)

ncols <- ncol(tbl)

rho <-0

if(ncols == 1 || nrows ==1)rho <- 0 else{

for(i in 1:nrows){

for(j in 1:ncols){

if ((sum(tbl[i,]) * sum(tbl[,j])==0)) r <- 0 else

r = tbl[i,j]^2/(sum(tbl[i,]) * sum(tbl[,j]))

rho = rho + r

}

}

}

return(rho -1)

}

The arguments of function are:

x = genotype for number of reference alleles

y = corresponding population membership

1. R code for LEI_freq function

lei_freq <- function(k,frq_pop,count_pop){

# k = number of populations

# frq_pop = arguments exclusively either MAF or Ref allele frequencies;vector length k

# count_pop = corresponding population counts; vector length k

if (k%%1!=0) stop("must be an integer")

if (!all(frq_pop>=0 & frq_pop<=1)) stop("all components must belong to [0,1]")

if (length(frq_pop)!=k) stop("vector length must be k")

if (!all(count_pop%%1==0)) stop("all components must belong to be integers")

if (length(count_pop)!=k) stop("vector length must be k")

n <- sum(count_pop)

tbl <- matrix(0, nrow = k, ncol = 3)

for (i in 1:k) {

f_a <- 1 - frq_pop[i]

t_1 <- count_pop[i]*c(frq_pop[i]^2, 2*frq_pop[i]*f_a, f_a^2)

tbl[i,] <- t_1

}

nrows <- k

ncols <- 3

rho <-0

for(i in 1:nrows){

for(j in 1:ncols){

if ((sum(tbl[i,]) * sum(tbl[,j])==0)) r <- 0 else

r = tbl[i,j]^2/(sum(tbl[i,]) * sum(tbl[,j]))

rho = rho + r

}

}

return(rho - 1)

}

The arguments of function are:

k = number of populations

frq_pop = vector of allele frequencies (length must be equal to k, each element corresponds to the population allele frequency: exclusively either MAF or Ref allele)

count pop = vector of population counts (length must be equal to k)

**Hardy-Weinberg Equilibrium (HWE)**

HWE is a mathematical equation that can be used to calculate the genetic variation of a population at equilibrium. The HWE states that the amount of genetic variation in a population remains constant from one generation to the next in the absence of disturbing factors (i.e., allele and genotype frequencies remain constant from generation to generation, a large random mating population with no genetic drift, no mutation, no migration, and no natural selection) ^1,2^. In a simple genetic locus at which there are two alleles, “A_1_” and “A_2_” with frequencies $p$ and $q$ respectively, the HWE is expressed as$: p^{2} + 2pq + q^{2} = 1$, where $p^{2}$ represents the frequency of the homozygous genotype A_1_A_1_, $q^{2}$ represents the frequency of the homozygous genotype A_2_A_2_, and $2pq$ represents the frequency of the heterozygous genotype A_1_A_2_. In addition, the sum of the allele frequencies for all the alleles at the locus must be 1, so $p + q = 1$. If the $p$ and $q$ allele frequencies are known, then the frequencies of the three genotypes can be calculated using the Hardy-Weinberg equation.

***Calculating allelic frequencies from genotypic frequencies***

Many methods in genetic studies such as population history studies, linkage and association analysis, calculation of linkage disequilibrium and admixture mapping require accurate estimates of allele frequencies. Allele frequencies are determined by calculating the relative proportion of an allele on a locus in a population ^2,3^. Inaccurate estimation of allele frequencies can cause false positives or reduce power in linkage analysis and lead to spurious or missed effects in association analysis and linkage disequilibrium calculation. Similarly, admixture mapping with case-only study design also needs strong prior information on the ancestral allele frequencies ^4^.

Suppose we have genotype data for $N$ randomly sampled individuals from a large population with two alleles (A_1_ and A_2_) at a locus with frequencies $p$ and $q \left( =1-p \right),$ respectively. We assume the frequencies of all genotypes (A_1_A_1_, A_1_A_2_, and A_2_A_2_) are known in the Table below. The estimator of the A_1_ allele frequency, $p$, is given by $p = (the number of A_{1})/2N$. Similarly, the frequency of the A_2_ allele, $q$, is given by $q = (the number of A_{2})/2N$. Then, based on the Table below, $p =\frac{2\left( count of A_{1}A_{1} \right)+count of A_{1}A_{2}}{2N}=\frac{2n_{11}+n_{12}}{2N}$ and $q = \frac{count of A_{1}A_{2}+2(count of A_{2}A_{2})}{2N}=\frac{n_{12}+2n_{22}}{2N}$ are the natural estimates of the frequencies of alleles A_1_ and A_2_.

Allele frequency estimation from genotype data

| Genotype | A_1_A_1_ | A_1_A_2_ | A_2_A_2_ | Total |
| --- | --- | --- | --- | --- |
| Genotype count | $n_{11}$ | $n_{12}$ | $n_{22}$ | $N$ |
| Frequency | $p_{11}$ | $p_{12}$ | $p_{22}$ | $1$ |

Given genotype data with two alleles in a locus from unrelated individuals that satisfy HWE assumptions (Table), the likelihood as a function of allele frequencies is given by:

$L\left( p;N \right)= \binom{N}{n_{11}, n_{12}, n_{22}}p_{11}^{n_{11}}p_{12}^{n_{12}}p_{22}^{n_{22}}$

$=\binom{N}{n_{11}, n_{12}, n_{22}}\left( p^{2} \right)^{n_{11}}\left( 2pq \right)^{n_{12}}\left( q^{2} \right)^{n_{22}}$.

Then, we can obtain the maximum likelihood estimate of $p$ by solving the first derivative of the log-likelihood $\mathcal{l}\left( p;N \right)=\ln\left( L\left( p;N \right) \right)$ as shown below:

$\mathcal{l}\left( p;N \right)=\ln\left( L\left( p;N \right) \right)=2n_{11}\ln p+n_{12}\ln p+n_{12}\ln q+2n_{22}\ln q +C$

$=\left( 2n_{11}+n_{12} \right)\ln p+\left( 2n_{22}+n_{12} \right)\ln q+C$

$=\left( 2n_{11}+n_{12} \right)\ln p+\left( 2n_{22}+n_{12} \right)\ln(1-p)+C$,

$\frac{d\left[ \mathcal{l}\left( p;N \right) \right]}{dp}=\frac{2n_{11}+n_{12}}{p}-\frac{2n_{22}+n_{12}}{1-p}=0$ 🡺$p=\frac{2\left( n_{11}+n_{12} \right)}{2(n_{11}+n_{12}+n_{22})}=\frac{n_{11}}{N}+\frac{n_{12}}{2N}$,

which is the natural estimate of the allele frequency of A_1_ as described above.

***Calculating genotype frequencies from allele frequencies***

When a population meets HWE assumptions (i.e., allele and genotype frequencies remain constant from generation to generation), the genotype frequencies can be calculated from the allele frequencies. For example, in a locus with two alleles A_1_ and A_2_ with frequencies p and q, the genotype frequency of A_1_A_1_, A_1_A_2_, and A_2_A_2_ are $p^{2}$, $2pq$, and $q^{2}$ respectively.

**Wright’s Fixation Indices (F_ST_):**

Wright’s fixation indices (F_ST_) ^5^ measures the degree of differentiation among populations, which was potentially arising due to the genetic structure within populations. Since then, multiple estimates of F_ST_ were developed and used. Nei ^6,7^ developed an analogue, G_ST_, as a ratio of expected heterozygosity within and among populations, and we executed using the allele frequencies from three populations and ranked the markers.

Global F_ST_: Let p_1_, p_2_, and p_3_ be the allele frequencies of the reference allele in the three sub-populations X_1_, X_2_, X_3_ and let n_1_, n_2_, and n_3_ be the sample sizes of the random samples from the respective populations. The expected heterozygosity within the subpopulations are given by $H_{i}=2p_{i}(1-p_{i})$, i = 1, 2, 3. If $\bar{p}=n_{1}p_{1}+n_{2}p_{2}+n_{3}p_{3}$ be the combine allele frequency, then the total heterozygosity is given by $H_{T}=2\bar{p}(1-\bar{p})$. If $H_{S}=\frac{n_{1}H_{1}+n_{2}H_{2}+n_{3}H_{3}}{n_{1}+n_{2}+n_{3}}$ be the weighted sum of the sub-population heterozygosity, the global F_ST_ can be estimated as ^6,7^:

$F_{ST}=\frac{H_{T}-H_{S}}{H_{T}}$.

**References**

1 Holsinger, K. The Hardy-Weinberg Principle and estimating allele frequencies. [*http://www.uvm.edu/~rsingle/stat395/S04/papers/eeb348_hardy-weinberg.pdf*](http://www.uvm.edu/~rsingle/stat395/S04/papers/eeb348_hardy-weinberg.pdf) (2004).

2 Adrianto, I. & Montgomery, C. Estimating allele frequencies. *Methods Mol Biol* **850**, 59-76, doi:10.1007/978-1-61779-555-8_5 (2012).

3 Chang, C. C. *et al.* Second-generation PLINK: rising to the challenge of larger and richer datasets. *Gigascience* **4**, 7 (2015).

4 Hoggart, C. J., Shriver, M. D., Kittles, R. A., Clayton, D. G. & McKeigue, P. M. Design and analysis of admixture mapping studies. *Am J Hum Genet* **74**, 965-978, doi:10.1086/420855 (2004).

5 Wright, S. The genetical structure of populations. *Ann Eugen* **15**, 323-354 (1951).

6 Nei, M. Analysis of gene diversity in subdivided populations. *Proc Natl Acad Sci U S A* **70**, 3321-3323 (1973).

7 Nei, M. F-statistics and analysis of gene diversity in subdivided populations. *Ann Hum Genet* **41**, 225-233 (1977).
